# Impact of treated wastewater reuse in agriculture on the transfer of antimicrobial-resistant bacteria and genes to edible crops: a One Health perspective

**DOI:** 10.3389/fmicb.2025.1729855

**Published:** 2026-01-15

**Authors:** Anicia Gomes, Jesús López-Cañizares, Macarena Moreno-Candel, Alberto Martinez-Alonso, Ana Allende, Pilar Truchado

**Affiliations:** 1 LAQV-REQUIMTE, Department of Biology, Faculty of Sciences, University of Porto, Porto, Portugal; 2 UCIBIO-Applied Molecular Biosciences Unit and Associate Laboratory i4HB—Institute for Health and Bioeconomy, Laboratory of Microbiology, Faculty of Pharmacy, University of Porto, Porto, Portugal; 3 Research Group on Microbiology and Quality of Fruit and Vegetables, CEBAS-CSIC (Spanish National Research Council), Murcia, Spain

**Keywords:** antimicrobial resistance, environmental surveillance, fresh produce, irrigation water, qPCR, sustainable agriculture, wastewater, water reuse

## Abstract

This study evaluated whether irrigation with treated wastewater of different microbiological quality (secondary- and tertiary-treated wastewater) contributes to the transmission of antibiotic-resistant bacteria (ARB) and antimicrobial resistance genes (ARGs) from irrigation water to lettuce plants, using potable water as control. Bacterial indicators (*Escherichia coli* and extended-spectrum β-lactamase-producing *E. coli*, ESBL-*E. coli*) and ARGs (*bla*_*CTX*–*M*–1_, *bla*_*TEM*_, *sul1*, *tetA*) were quantified in irrigation water and lettuce using culture-based methods and quantitative PCR (qPCR). In addition, the efficiency of tertiary treatment in reducing *Escherichia coli*, ESBL-*E. coli*, and resistance genes in reclaimed water was assessed. The relative abundance of ARGs was normalized to the 16S rRNA gene to evaluate potential amplification or persistence of resistance during water reuse and irrigation. Results showed that *E. coli* and ESBL-*E. coli* were consistently detected in crops irrigated with secondary-treated water but remained below detection limits after irrigation with tertiary-treated and potable water. Resistance gene profiles followed a similar trend: secondary-treated water contained the highest absolute and relative abundances of *bla*_*CTX*–*M*–1_, *bla*_*TEM*_, *sul1*, and *tetA*, while tertiary treatment substantially reduced but did not completely eliminate them. In lettuce, ARG levels on lettuce were substantially lower than in the corresponding irrigation waters, representing only 4 and 6% of the concentrations detected in tertiary- and secondary-treated wastewater, respectively. This reduction indicates limited transfer and/or persistence of ARGs on the plant surface despite detectable levels in the irrigation water. Our study provides valuable insights into the role of poor-quality irrigation water in driving ARGs dissemination to fresh produce and shows that advanced tertiary treatments significantly reduce AMR-related risks, thereby supporting the safe and sustainable use of reclaimed water in agriculture.

## 1 Introduction

Water scarcity and the growing demand for sustainable food production are major global challenges. These pressures are driving the need to develop water management plans and use alternative water sources (Garner et al., 2021; Seyoum et al., 2022). The Food and Agriculture Organization (FAO) estimates that 1,660 million ha of land, corresponding to more than 10% of the world’s land area, is degraded due to the impact of human activities (Food and Agriculture Organization [FAO] of the United Nations, 2025), underscoring the urgency of developing innovative water reuse strategies.

Globally, the reuse of treated and untreated wastewater for agricultural irrigation is a widespread and expanding practice. It is currently applied in more than 50 countries and covers over 20 million hectares of farmland, particularly in water-scarce regions such as Asia, North Africa, the Middle East, the United States and southern Europe (Mishra et al., 2023). This global increase reflects the growing reliance on wastewater as an alternative water source to sustain crop production under increasing water scarcity. Effluents from urban wastewater treatment plants (WWTP), such as reclaimed water, are a readily available resource that the European Commission (EC) already promotes for agricultural irrigation to alleviate pressure on freshwater supplies (European Commission, 2025). However, the use of reclaimed water in crop production can pose a risk of contaminating fresh produce with foodborne pathogens, ARB and ARGs, mostly because these products are consumed raw (Berendonk et al., 2015; Seyoum et al., 2022). Wastewater treatment plants receive water from multiple sources and have been identified as potential hotspots for the dissemination of ARB and ARGs. Wastewater often contains high bacterial loads and may contain antibiotic residues, and the treatment processes used in WWTPs typically create conditions that promote interactions among millions of bacteria, facilitating bacterial selection and the persistence of ARGs within water microbial communities (Hazra et al., 2024; Machado et al., 2023). Considering that, according to the World Health Organization (World Health Organization [WHO], 2023), antimicrobial resistance was directly responsible for approximately 1.27 million deaths and associated with nearly 5 million deaths worldwide in 2019. In this context, the potential dissemination of ARBs and ARGs through reclaimed water constitutes a major global public-health concern.

The current European regulation on minimum requirements for water reuse in agriculture [Regulation (EU) 2020/741] sets specific minimum microbiological criteria, but it also indicates that a risk assessment should be performed to determine if other potential hazards, such as antimicrobial resistant determinants, might represent a potential risk (European Commission, 2020). With the aim of gaining knowledge about the potential of ARB and ARGs to persist in treated effluents and disseminate into crops, more studies should focus on the monitorization of ARGs through the water reuse system. Previous studies have demonstrated the capacity of WWTP to effectively reduce the abundance of ARB (Manaia et al., 2018; Bonetta et al., 2023; Macrì et al., 2024), however, ARGs are still frequently detected in treated effluents (Wang et al., 2020; Park et al., 2024). Therefore, when reclaimed water is used for crop irrigation, these effluents may introduce ARGs into cultivated crops, promoting their dissemination and raising public health concerns (Cacace et al., 2019; Marano et al., 2019). A major challenge arises from the detection of resistance genes to last-resort antibiotics, such as extended-spectrum β-lactamase (ESBL) genes (Hembach et al., 2017).

Several studies have already evaluated the transmission of ARG from water to crops, but their findings remain inconsistent. Some authors report that irrigation with treated wastewater increases the abundance and diversity of ARGs in soils and edible plant tissues (Wang et al., 2014; Han et al., 2016). In contrast, other studies have found no significant differences between crops irrigated with treated wastewater and those irrigated with freshwater, suggesting that environmental conditions, soil properties, crop type, and irrigation practices may strongly influence ARG transfer (Orlofsky et al., 2016; Marano et al., 2019; Seyoum et al., 2021, 2022). Given these concerns, this study aimed to evaluate whether the use of treated wastewater (secondary and tertiary treated wastewater) contributes to the transmission of ARB and ARGs from water to lettuce grown under controlled conditions (growing chambers). Specifically, the study focuses on the detection of *Escherichia coli*, extended-spectrum β-lactamase (ESBL)-producing *E. coli*, and selected ARGs encoding resistance to β-lactams, sulfonamides, and tetracyclines.

## 2 Materials and methods

### 2.1 Experimental design

Experiments were conducted under controlled environmental conditions in a growth chamber simulating those of a commercial greenhouse, with a 12-h light and 12-h dark photoperiod, temperatures ranging from 18 to 23°C, and 75% relative humidity. Two-week-old lettuce seedlings were distributed into three experimental conditions, each consisting of four trays with 234 plantlets per tray, which were subsequently pooled for analysis. To assess the potential transmission of ARB and ARGs from water to crops, lettuce plants were exposed to three different irrigation regimes: (1) potable water (municipal tap water, control), spray-irrigated every 2 days until the end of the experiment (day 15); (2) secondary treated wastewater, spray-irrigated every 2 days; and (3) tertiary treated wastewater, spray-irrigated every 2 days using reclaimed chlorine-disinfected water. For clarity, throughout the manuscript, these treatments are referred to as “potable water” (control), “secondary-treated water” (secondary), and “tertiary-treated water” (tertiary). Experiments were carried out twice to ensure repeatability of the assays (*n* = 2).

### 2.2 Irrigation water source and sampling

Treated wastewater was collected from a WWTP located in the Region of Murcia (Spain), which applies aeration, solids and suspended solids separation, grit removal, and degreasing during primary treatment; a double-stage activated sludge process with coagulation/flocculation and lamella clarification during secondary treatment; and sand filtration and UV-C disinfection as tertiary treatment. Water samples from the treatment plant were collected at least twice for each independent assay to avoid significant decreases in the concentration of ARB or ARGs. Water samples (4–6 L) were collected on days 0 and 10. In total, six samples per experimental condition were obtained. Samples were transported under refrigeration (≤ 2 h) to the laboratory, where 2 L was transferred into sterile polypropylene bottles (Labbox Labware S.L., Barcelona, Spain) and stored at 4°C for further analysis. Potable water, collected at the same time points, was included as a negative control and handled under identical conditions.

Lettuce plants were sampled at four time points: day 0, before the start of the experimental irrigation regime to establish baseline levels of antimicrobial resistance genes; and days 5, 10, and 15, corresponding to the period until plants reached commercial maturity, defined in this study as a size of approximately 15 cm measured from the petiole, consistent with previous descriptions of commercial maturity in baby leafy vegetables (Truchado et al., 2017). At each time point, three replicates of 100 g of lettuce were randomly harvested from different trays to minimize positional bias, using sterile scissors to cut at the base of the petiole. Samples were placed in sterile Stomacher filter bags.

### 2.3 Water microbiological analysis

Irrigation water was analyzed for the enumeration of *E. coli* and ESBL-producing *E. coli*. To account for differences in bacterial load, samples were processed in duplicate using different volumes (1, 10, and 100 mL) by membrane filtration through 0.45 μm cellulose nitrate filters (Sartorius, Madrid, Spain) with a manifold system (Millipore, Madrid, Spain). The filters were transferred onto CHROMagar™ and Chromocult^®^ agar plates and incubated at 37°C for 24 h. In addition, 10-fold serial dilutions (10^0^ to 10^–3^) of potable water, secondary-treated wastewater, and tertiary-treated wastewater were prepared in buffered peptone water (2 g/L), and 100 μL of each dilution was plated in duplicate on the same media. Plates were incubated for 24 h at 37°C before result interpretation. Dark blue–violet colonies were considered positive for *E. coli*, and dark pink to reddish colonies were interpreted as ESBL-producing *E. coli*. Colony counts were expressed as CFU/100 mL, and the limit of detection (LOD) was estimated based on the highest analyzed volume. For all water samples, the LOD corresponded to 1 CFU/100 mL. The limit of quantification (LOQ) was 5 CFU per plate (5 CFU/100 mL).

### 2.4 Lettuce sampling and microbiological analysis

For microbiological analysis of lettuce, samples of 50 g were taken on days 5, 10, and 15 (D5, D10, D15) of the assays and placed in Stomacher bags containing buffered peptone water (BPW, 2 g/L) supplemented with 0.1% Tween 80, at a ratio of 1:9 (w/v). Bags were gently shaken for 1 min to detach surface bacteria without disrupting the tissue, and the resulting leaf washes were used for microbiological determinations. Two independent assays were conducted following this procedure. *Escherichia coli* and ESBL-producing *E. coli* were analyzed using Chromocult^®^ and CHROMagar™ media, respectively. To account for differences in bacterial load, different sample volumes (1, 10, and 100 mL) of the leaf wash were processed in duplicate by membrane filtration through 0.45 μm cellulose nitrate filters (Sartorius, Madrid, Spain) using a manifold system (Millipore, Madrid, Spain). The filters were then placed onto the corresponding media and incubated at 37°C for 24 h. After incubation, dark blue–violet colonies and dark pink to reddish colonies were considered positive for *E. coli* and ESBL-producing *E. coli*, respectively. A total of 18 samples were analyzed per treatment. Colony counts were expressed as CFU/g. For lettuce samples, the LOD corresponded to 1 CFU in the filtered volume (100 mL), equivalent to 0.08 CFU/g. The limit of quantification (LOQ) corresponded to 5 CFU per membrane, equivalent to 0.4 CFU/g.

### 2.5 Sample concentration and DNA extraction

Water samples were also concentrated for DNA extraction by filtering 6 L of potable water, 1 L of tertiary-treated water, and 400 mL of secondary-treated water through nitrocellulose filters with a 0.22 μM pore size. Different volumes were filtered based on the expected microbial load, with less volume needed for secondary-treated water due to its higher biomass and larger volumes required for potable and tertiary-treated water to obtain sufficient DNA. Each filter was placed into a 50 mL Falcon tube containing 20 mL of BPW with 0.1% Tween 80 and briefly vortexed to recover the material retained on the filter. The filter was then removed with a sterile loop. Tubes were centrifuged at 3,000 × g for 10 min, the supernatant discarded, and the pellet transferred to a 1.5 mL tube. The pellet was centrifuged again at 9,000 × g for 10 min, the supernatant discarded, and the pellet stored at −*20*°C until DNA extraction.

For lettuce samples, 100 mL of the leaf wash suspension were filtered through 0.22 μm membranes, which were subsequently placed into 50 mL Falcon tubes containing 20 mL of BPW supplemented with 1% Tween 80, briefly vortexed, and removed before centrifugation. The centrifugation steps were identical to those described for water samples, and the resulting pellets were stored at −*20*°C until DNA extraction. Total DNA was extracted from the sample pellets using the DNeasy PowerSoil Pro Kit (QIAGEN^®^), following the manufacturer’s instructions. DNA concentrations and purity were determined by an Implen NanoPhotometer N60/50 (Implen, Munich, Germany). The DNA samples were then stored at −*20*°C.

### 2.6 Antimicrobial resistance genes and qPCR assay

Quantitative PCR (qPCR) was used to quantify the total bacterial population through the 16S rRNA gene. In this study, 16S rRNA measurements represent genomic copies per 100 mL (water) or per gram (plant tissue) and were used as an indicator of total bacterial gene abundance. Additionally, the presence and quantification of ARGs conferring resistance to β-lactams (*bla*_CTX–M–1_ and *bla*_TEM_), sulfamethoxazole (*sul1*), and tetracycline (*tetA*) were also assessed. The selection of *bla*_CTX–M–1_ *bla*_TEM_, *sul1* and *tetA* was based on their identification as key environmental AMR surveillance markers in both the qPCR framework proposed by Keenum et al. (2022) and the priority resistance determinants highlighted by EFSA Panel on Biological Hazards (BIOHAZ) et al. (2021).

The quantification assays were performed using a QuantStudio 5 system (Applied Biosystems, United States) in 96-well plates with KAPA SYBR FAST and KAPA PROBE FAST Universal qPCR Master Mix kits (KapaBiosystems, Massachusetts, United States). The primers and probes used to quantify the ARGs, as well as the reaction conditions, are listed in Table 1. All reactions were run in triplicate, with positive and negative controls (nuclease-free water) included in each run, and performed in a final volume of 20 μL. Standard curves were generated from seven- to eight-point, 10-fold serial dilutions of genomic DNA obtained from reference bacterial strains, following the procedure described by Truchado et al. (2016). The strains used were *E. coli* SL6.1 (University of Porto, Portugal) for *bla*_CTX–M–1_ quantification, *Klebsiella pneumoniae* 997156 (University of Granada, Spain) for *bla*_TEM_ and *sul1* quantification, and *E. coli* CIP 130470 (Pasteur Institute, Paris, France) for 16S rRNA and *tetA* quantification. The LOD was established from tenfold serial dilutions of DNA standards and defined as the lowest concentration consistently amplified in ≥ 95% of replicates. LODs varied by sample matrix. For water samples, LODs were 1.62 (*bla*_CTX–M–1_), 0.35 (*bla*_TEM_), 1.30 (*sul1*), 1.75 (*tetA*) and 1.33 (16S rRNA), log_10_ genomic copies (gc) per 100 mL. In plant tissue, LODs were 1.01 (*bla*_CTX–M–1_), 0.85 (*bla*_TEM_), 1.98 (*sul1*), 0.96 (*tetA*) and 0.83 (16S rRNA) log_10_ gc per gram. Absolute abundances of ARGs (gc) were calculated using standard curves generated from genomic DNA of reference strains, while relative abundances Log (ARG/16S rRNA) were obtained by normalizing each ARG to the corresponding 16S rRNA gc. Thus, both absolute and relative quantification approaches were applied in this study.

**TABLE 1:** Primers and probes selected to quantify ARGs and the cycling parameters used in the qPCR reactions.

Target gene	Primers and probes (5’ → 3’)		Conditions	References
16S rRNA	F1048	GTGSTGCAYGGYTGTCGTCA	50°C for 2 min; 95°C for 3 min; 35 cycles of (95°C for 5 s, 60°C for 30 s, 72°C for 1 min	(Maeda et al., 2003)
	R1194	ACGTCRTCCMCACCTTCCTC		
*bla*_CTX–M–1_	FW	ACCAACGATATCGCGGTGAT	95°C for 10 min; 45 cycles of (95°C for 30 s, 60°C for 1 min)	(Colomer-Lluch et al., 2011)
	RV	ACATCGCGACGGCTTTCT		
	Probe	FAM-TCGTGCGCCGCTG-MGBNFQ		
*bla*_TEM_	FW	CATTTCCGTGTCGCCCTTATTC	50°C for 2 min; 95°C for 3 min; 40 cycles of (95°C for 5 s, 60°C for 30 s, 72°C for 1 min	(Dallenne et al., 2010)
	RV	CGTTCATCCATAGTTGCCTGAC		
*sul1*	FW	CCGTTGGCCTTCCTGTAAAG	95°C for 10 min; 45 cycles of (95°C for 30 s, 58°C for 1 min)	(Ginn et al., 2021)
	RV	TTGCCGATCGCGTGAAT		
	Probe	FAM-CAGCGAGCCTTGCGGCGG-BHQ1		
*tetA*	FW	CCGCGCTTTGGGTCATT	95°C for 10 min; 45 cycles of (95°C for 30 s, 56°C for 1 min)	(Ginn et al., 2021)
	RV	TGGTCGCGTCCCAGTGA		
	Probe	FAM-TCGGCGAGGATCG-BHQ		

### 2.7 Statistical analysis

Counts of *E. coli* and ESBL-producing *E. coli* below the LOQ were excluded from log_10_ transformation and subsequent statistical analyses. For the calculation and graphical representation of the median and interquartile range (IQR), only positive samples, values above the LOQ were included. The prevalence of *E. coli* and ESBL-producing *E. coli* was considered positive when the sample was below the LOD. For ARGs, prevalence was defined as a sample being positive when at least two out of three qPCR wells showed amplification, even if the values were below the LOD. For absolute quantification, values below the LOD for each gene were excluded from statistical analyses. For relative abundance, log_10_ (ARG/16S rRNA) values below the LOD were replaced with the LOD value to allow ratio computation and ensure consistency across samples. Statistical analyses were performed using IBM SPSS Statistics 29 (IBM, Armonk, NY, United States). Data normality was assessed using the Shapiro-Wilk test (*P* > 0.05). Since the data followed a normal distribution, a one-way analysis of variance (ANOVA) was applied to determine whether statistically significant differences existed among the samples (*P* ≤ 0.05), followed by Tukey’s HSD multiple comparison test. Different groups were indicated with distinct letters (a, b, c). Unless otherwise stated, *P*-values ≤ 0.05 were considered statistically significant. A heatmap illustrating the relative abundance [log_10_ (ARG/16S rRNA)] of ARGs in water sources and lettuce samples was generated using R software (R Core Team, 2021) with the ggplot2 package (Wickham, 2016).

## 3 Results

### 3.1 Prevalence and enumeration of *E. coli* and ESBL-producing *E. coli* in irrigation water and crops

**FIGURE 1:**
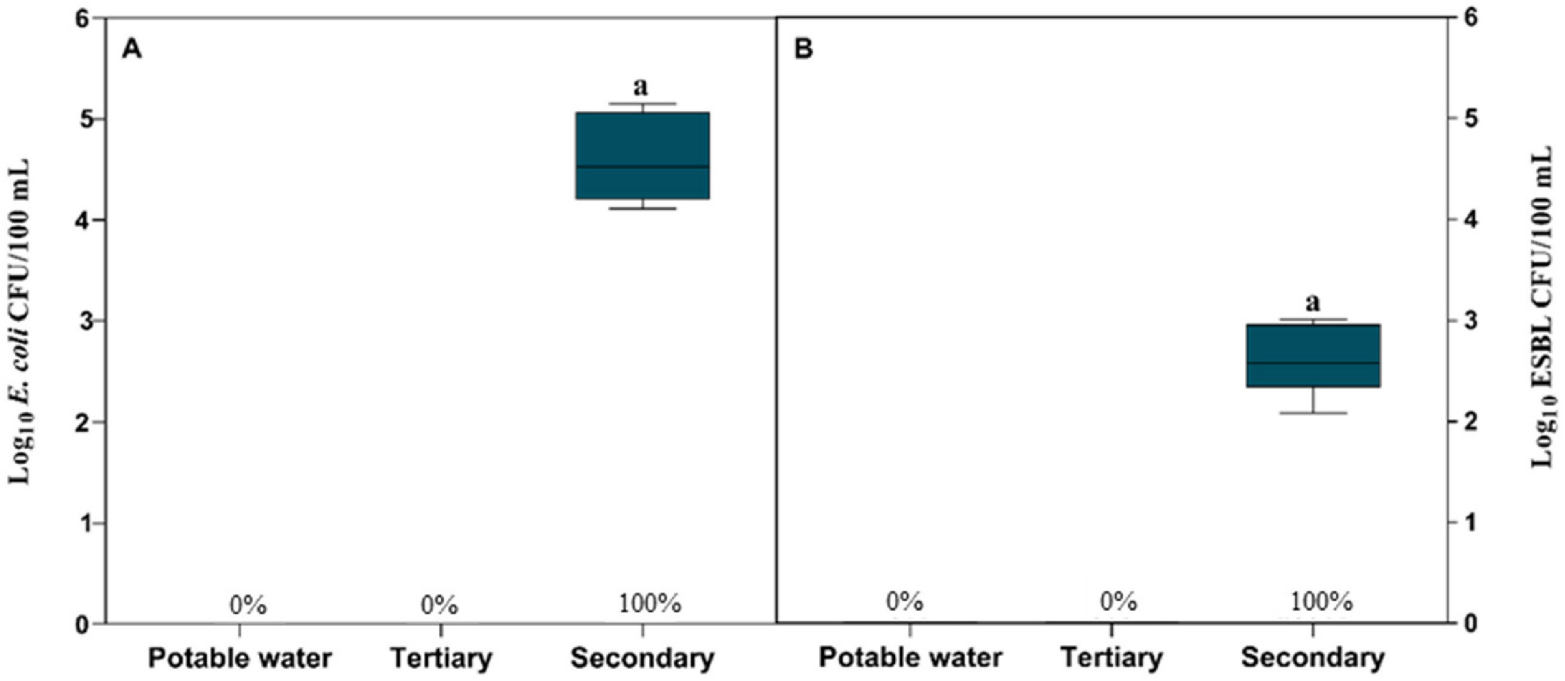
Abundance of *E. coli* **(A)** and ESBL-producing *E. coli* **(B)** in potable water, tertiary- and secondary-treated wastewater used for irrigation during the lettuce growing cycle. Results are expressed as Log CFU/100 mL. Box plots represent the median and the 25th–75th percentile values. Box plots labeled with different lowercase letters indicate statistically significant differences among treatments at *P* < 0.05. The percentage indicates the prevalence of *E. coli* and ESBL-producing *E. coli*, respectively, in each sample.

The enumeration of *E. coli* and ESBL-producing *E. coli* in irrigation water revealed significant differences among treatments (Figure 1). Both potable water and tertiary-treated water were below the LOD for *E. coli*, whereas secondary-treated water showed detectable levels in all samples (100%, *n* = 18), with a mean concentration of 4.59 ± 0.44 log_10_ CFU/100 mL. ESBL-producing *E. coli* also remained below the LOD in potable and tertiary-treated water but was detected in 100% (*n* = 18) of secondary-treated water samples, with a mean abundance of 2.61 ± 0.33 log_10_ CFU/100 mL. These results demonstrate the substantially higher prevalence and abundance of both *E. coli* and ESBL-producing *E. coli* in secondary-treated water and confirm the effectiveness of tertiary treatment in reducing microbial loads to non-detectable levels.

**FIGURE 2:**
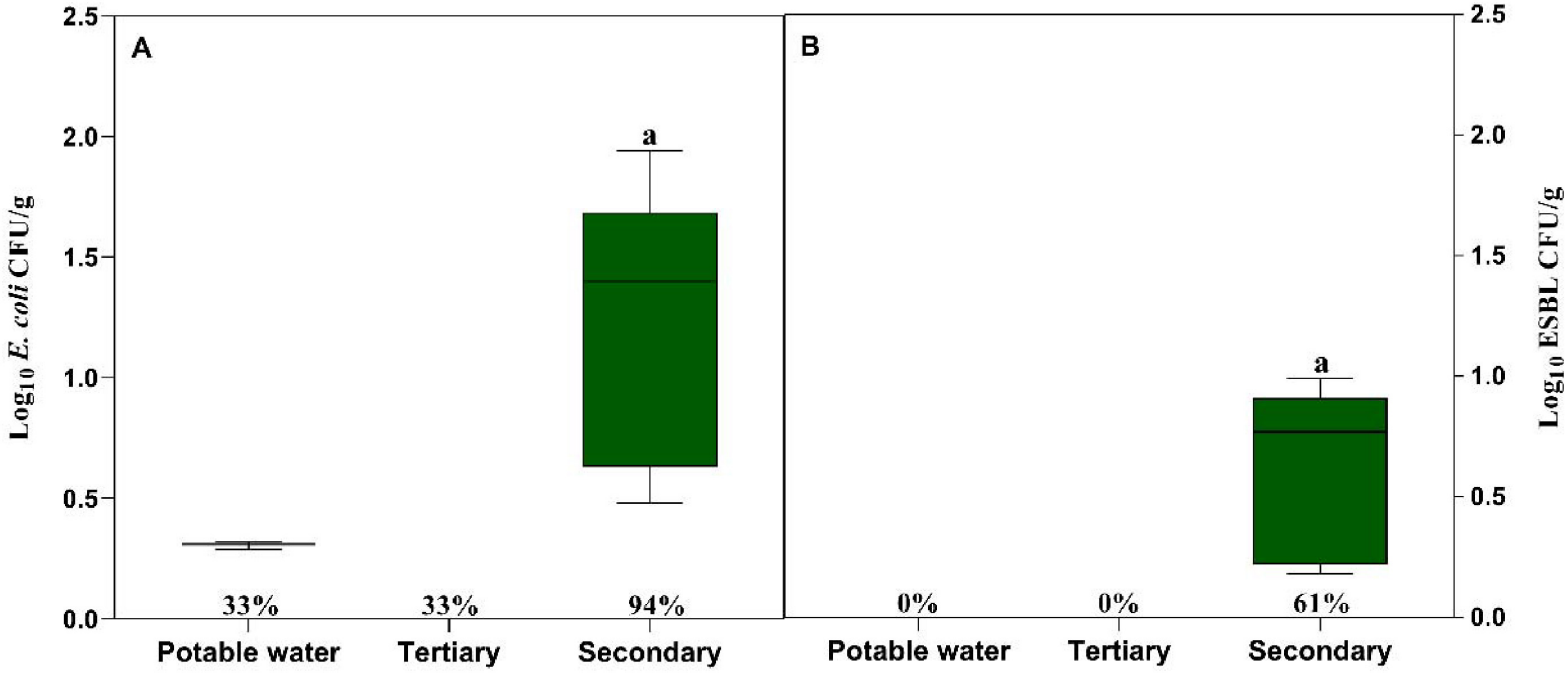
Abundance of *E. coli* **(A)** and ESBL-producing *E. coli* **(B)** on lettuce samples irrigated with potable water, tertiary-treated water, and secondary-treated water used for irrigation during the lettuce growing cycle. Results are expressed as Log CFU/g fresh weight. Box plots represent the median and the 25th–75th percentile values. Box plots labeled with different lowercase letters indicate statistically significant differences among treatments at *P* < 0.05. The percentage indicates the prevalence of *E. coli* and ESBL-producing *E. coli*, respectively, in each sample.

In lettuce plants irrigated with different water qualities (Figure 2), *E. coli* was detected in 33.3% (*n* = 6) of plants irrigated with potable water and in 33.3% (*n* = 6) of those irrigated with tertiary-treated water, whereas a substantially higher prevalence (94%, *n* = 17) was observed in plants irrigated with secondary-treated water. Mean *E. coli* abundances were below the LOQ in lettuce irrigated with potable and tertiary-treated water and reached 1.26 ± 0.51 log_10_ CFU/g in lettuce irrigated with secondary-treated water. Extended-spectrum β-lactamase (ESBL)-producing *E. coli* was below the LOD in plants irrigated with potable or tertiary-treated water but was detected in 61% (*n* = 11) of plants irrigated with secondary-treated water, with a mean abundance of 0.61 ± 0.39 log_10_ CFU/g. Although the ESBL-producing *E. coli* counts showed wide dispersion among individual plants, particularly in those irrigated with secondary-treated water, the overall trend indicates that this treatment led to both a higher prevalence of ESBL-producing *E. coli* and increased microbial loads. In contrast, tertiary-treated water consistently maintained both *E. coli* and ESBL-producing *E. coli* below the LOD.

### 3.2 Levels of 16S rRNA in irrigation water and crops

**FIGURE 3:**
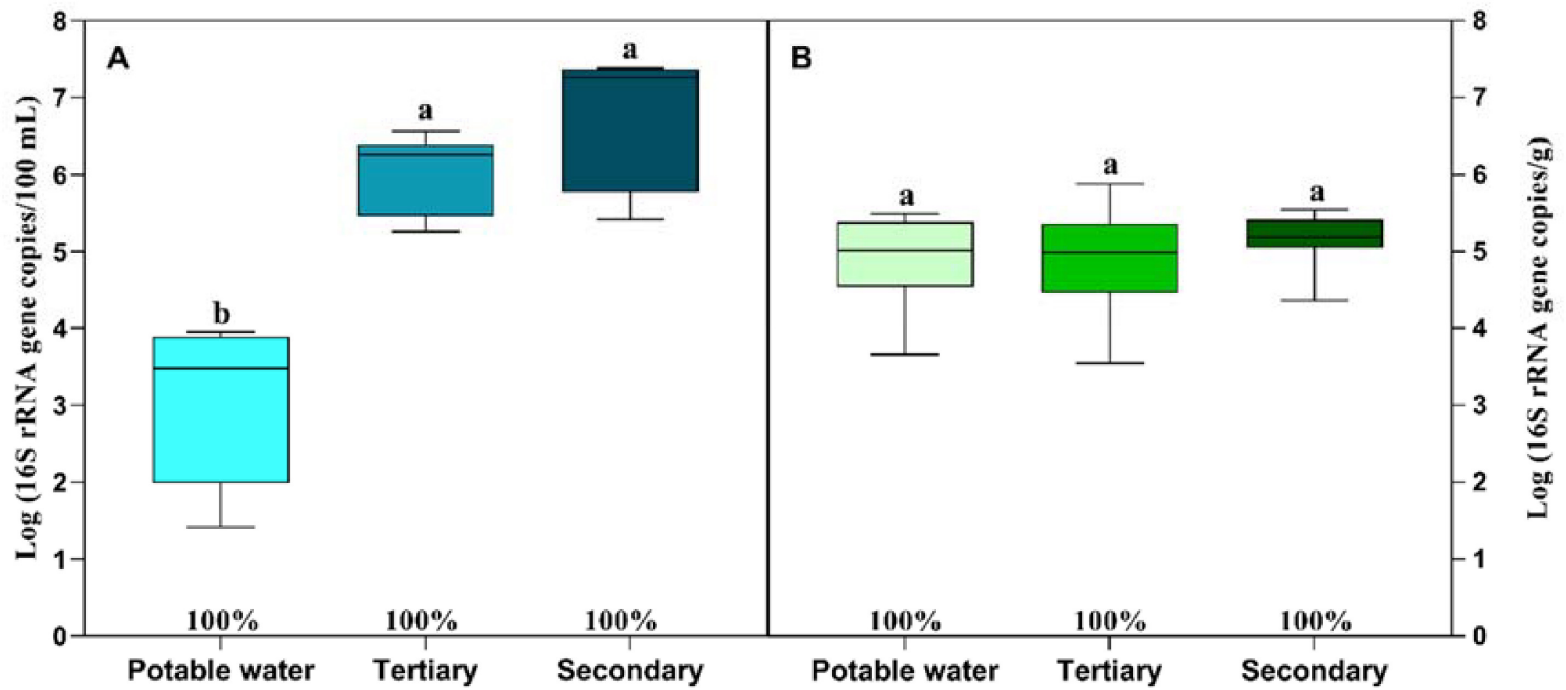
Absolute abundance of 16S rRNA in potable water, tertiary-treated water, and secondary-treated water used for irrigation during the lettuce growing cycle **(A)** and in lettuce plants irrigated with the same water samples throughout the growing cycle **(B)**. Box plots represent the median and the 25th–75th percentile values. Box plots labeled with different lowercase letters indicate statistically significant differences among treatments at *P* < 0.05. The percentage indicates the presence of 16S rRNA in each sample. Data correspond to trials 1 and 2 (*n* = 18).

The abundance of the 16S rRNA gene in water samples varied markedly depending on the source (Figure 3A). Potable water showed the lowest bacterial load, while tertiary and secondary treated water exhibited significantly higher bacterial abundances. In lettuce, 16S rRNA gene levels were similar across all irrigation treatments (Figure 3B), with no significant differences observed among plants irrigated with potable, tertiary or secondary treated water.

### 3.3 Prevalence and absolute levels of ARGs detected in water samples

**FIGURE 4:**
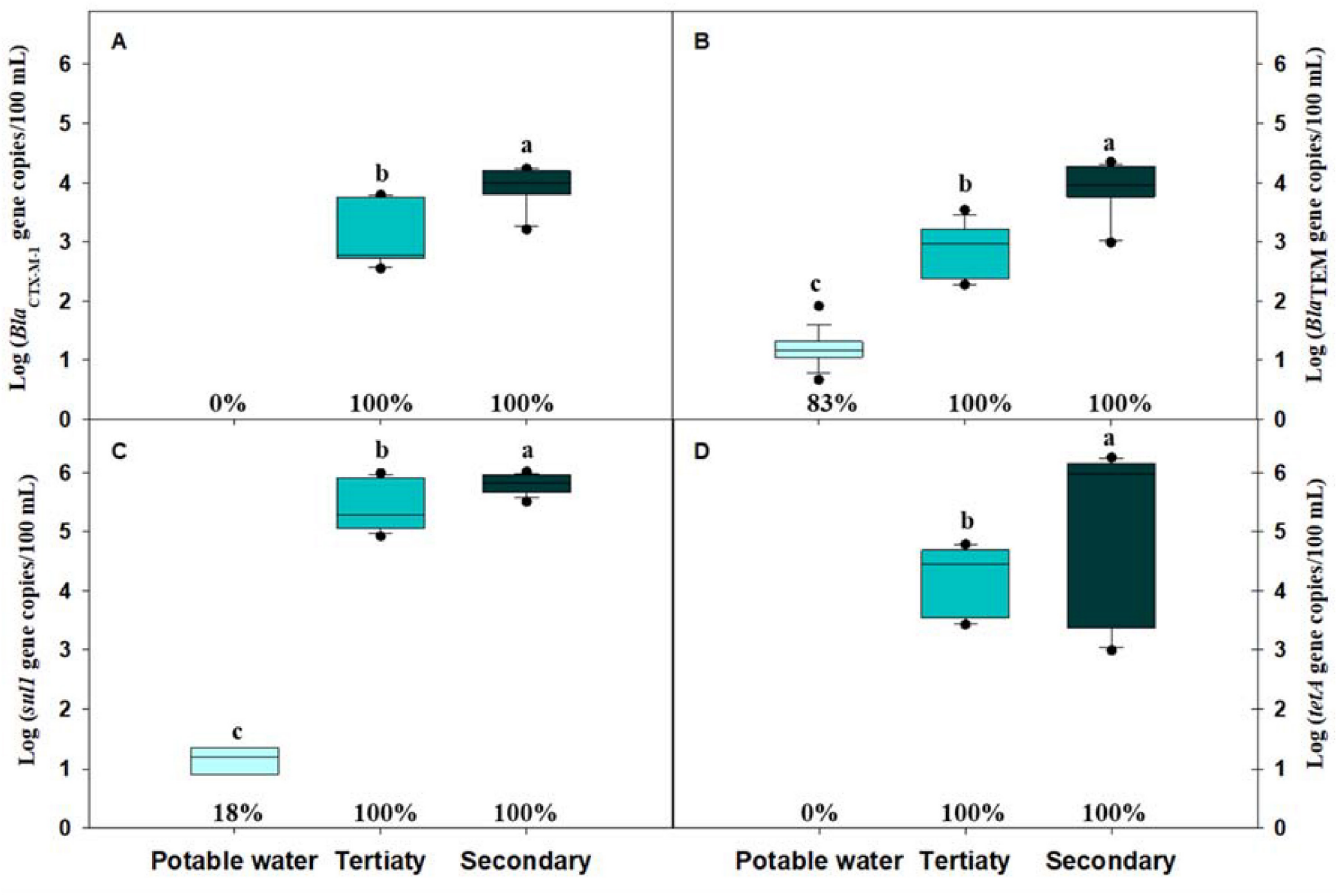
Absolute concentrations of four antibiotic resistance genes (ARGs) in potable water, tertiary-treated water, and secondary-treated water used for irrigation during the lettuce growing cycle. Box plots represent the median and the 25th–75th percentile values. Box plots labeled with different lowercase letters indicate statistically significant differences among treatments at *P* < 0.05. The percentage indicates the prevalence of ARGs in each sample. **(A)** *bla*_CTX–M–1_; **(B)** *bla*_TEM_; **(C)** *sul1*; **(D)** *tetA*. Data correspond to trials 1 and 2 (*n* = 18).

The prevalence of the four target ARGs (*bla*_CTX–M–1_, *bla*_TEM_, *sul1*, and *tetA*) differed markedly among the three irrigation water sources (Figure 4). The absolute abundance of these genes also varied significantly across treatments. Potable water presented the lowest ARG levels, with *sul1* at log_10_ 0.99 ± 0.35 gc/100 mL (18%; *n* = 1), *bla*_TEM_ at log_10_ 1.18 ± 0.28 gc/100 mL (83%; *n* = 5), while *tetA* and *bla*_CTX–M–1_ were consistently below their LODs (1.62 and 1.75 log_10_ gc/100 mL) respectively. In tertiary-treated water, all four genes showed 100% (*n* = 9) prevalence. Tertiary-treated water displayed significantly lower values than secondary-treated water for *sul1* and *tetA* (log_10_ 5.12 ± 1.40 gc/100 mL and log_10_ 4.25 ± 0.55 gc/100 mL, respectively). *bla*_TEM_ and *bla*_CTX–M–1_ were detected at mean concentrations of log_10_ 2.87 ± 0.43 gc/100 mL and log_10_ 3.07 ± 0.51 gc/100 mL, respectively. Secondary-treated water exhibited the same prevalence (100%, *n* = 18, for all genes) and the highest absolute abundances, with mean concentrations of log_10_ 5.80 ± 0.15 gc/100 mL for *sul1*, log_10_ 5.12 ± 1.40 gc/100 mL for *tetA*, log_10_ 3.92 ± 0.33 gc/100 mL for *bla*_CTX–M–1_ and log_10_ 3.89 ± 0.33 gc/100 mL for *bla*_TEM_. Overall, secondary-treated water showed the highest ARG abundances, tertiary-treated water showed intermediate levels, and potable water showed minimal contamination.

### 3.4 Prevalence and absolute levels of ARGs detected in crops

**FIGURE 5:**
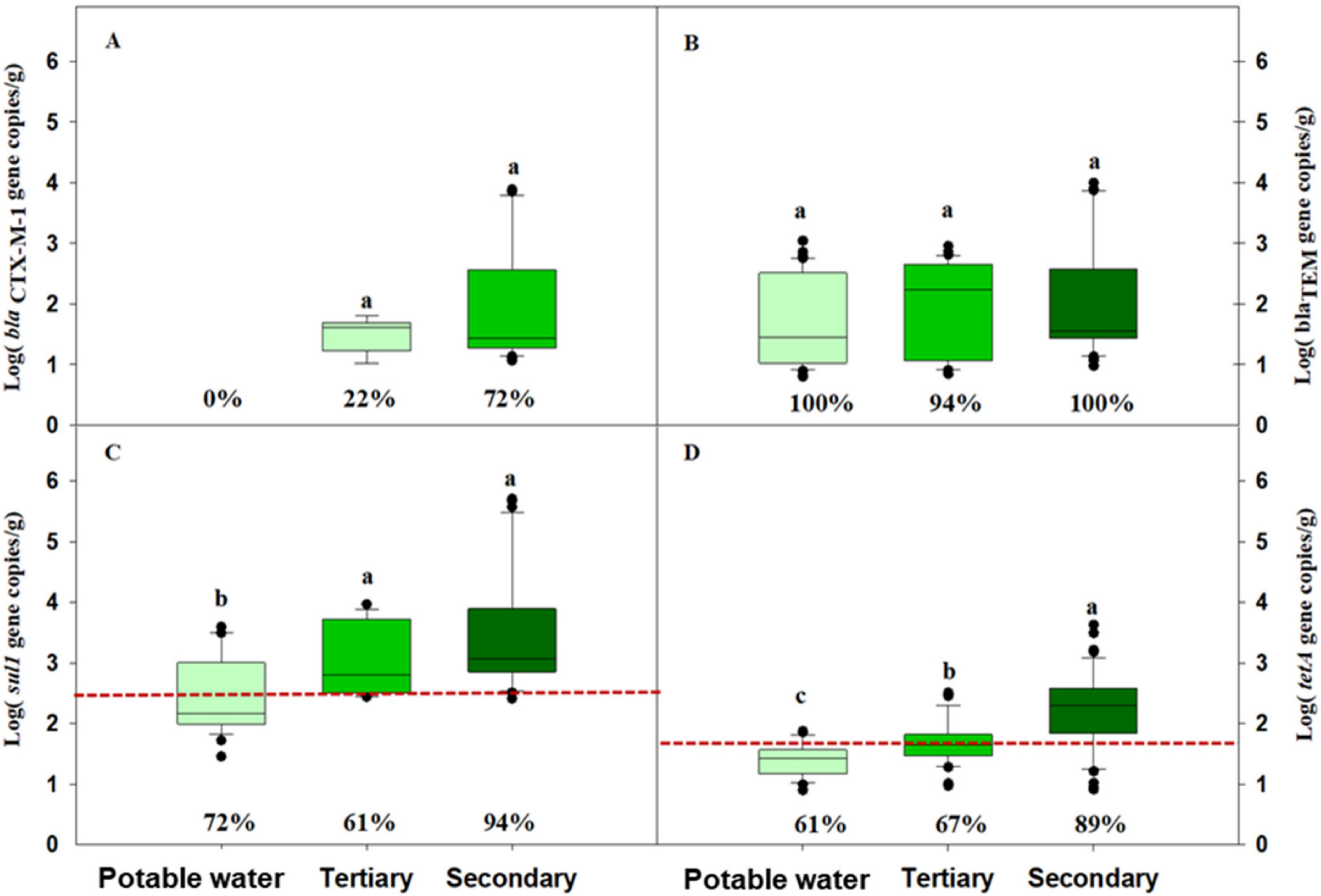
Absolute concentrations of four antibiotic resistance genes (ARGs) in lettuce plants irrigated with potable water, tertiary-treated, and secondary-treated water throughout the growing cycle. Box plots show the median and the 25th–75th percentile values. Box plots marked with different lowercase letters indicate statistically significant differences among treatments at *P* < 0.05. Percentages denote the prevalence of ARG detection in each sample. The red dotted line indicates the baseline levels of ARGs detected in lettuce plants. **(A)** *bla*_CTX–M–1_; **(B)** *bla*_TEM_; **(C)** *sul1*; **(D)** *tetA*. Data correspond to trials 1 and 2 (*n* = 18).

The prevalence of detection for the four ARGs analyzed (*bla*_CTX–M–1_, *bla*_TEM_, *sul1*, and *tetA*) in lettuce plants irrigated with the three tested water sources is shown in Figure 5. At baseline (d 0), *sul1* and *tetA* were detected, while *bla*_CTX–M–1_ and *bla*_TEM_ were below LODs (Table 2). Overall, plants irrigated with secondary-treated water exhibited the highest prevalence for most genes, followed by those irrigated with tertiary-treated water, whereas the control group (potable water) displayed the lowest prevalence.

**TABLE 2:** Baseline concentrations of 16S rRNA and ARGs in baby lettuce before the irrigation treatments.

ARGs	Log cg/g
16S rRNA	5.61 ± 1.84
*Bla*_CTX–M–1_	No amplification (LOD 1.01)
*Bla*_TEM_	Amplification < LOD (0.85)
*sul1*	2.46 ± 0.20
*tetA*	1.80 ± 0.74

Values are expressed as log gene copies per gram (log cg/g). “No amplification” indicates values below the limit of detection (LOD); “Amplification < LOD” indicates detection but below the quantification threshold. Data correspond to trials 1 and 2 (*n* = 6).

Figure 5 also shows the absolute abundances of ARGs in lettuce irrigated with the different water sources. In plants irrigated with potable water all genes except *bla*_CTX–M–1_ were detected. Plants irrigated with tertiary-treated water exhibited intermediate gene abundances (2.63 ± 0.68 log_10_ gc/g for *sul1*, 1.65 ± 0.57 log_10_ gc/g for *tetA*, 1.95 ± 0.76 log_10_ gc/g for *bla*_TEM_ and 1.48 ± 0.28 log_10_ gc/g for *bla*_CTX–M–1_). Plants irrigated with secondary-treated water consistently showed the highest values for all genes: 3.26 ± 1.10 log_10_ gc/g for *sul1*, 2.34 ± 0.63 log_10_ gc/g for *tetA*, 2.03 ± 0.95 log_10_ gc/g for *bla*_TEM_, and 1.97 ± 0.30 log_10_ gc/g for *bla*_CTX–M–1_. Statistically significant differences in the absolute abundance of *sul1* and *tetA* were detected between lettuce irrigated with potable water and lettuce irrigated with secondary- or tertiary-treated water. For *tetA*, absolute abundances in plants irrigated with secondary-treated water were higher than in those irrigated with tertiary-treated water and in the control plants.

### 3.5 Relative abundance of ARGs in irrigation water and baby lettuce

**FIGURE 6:**
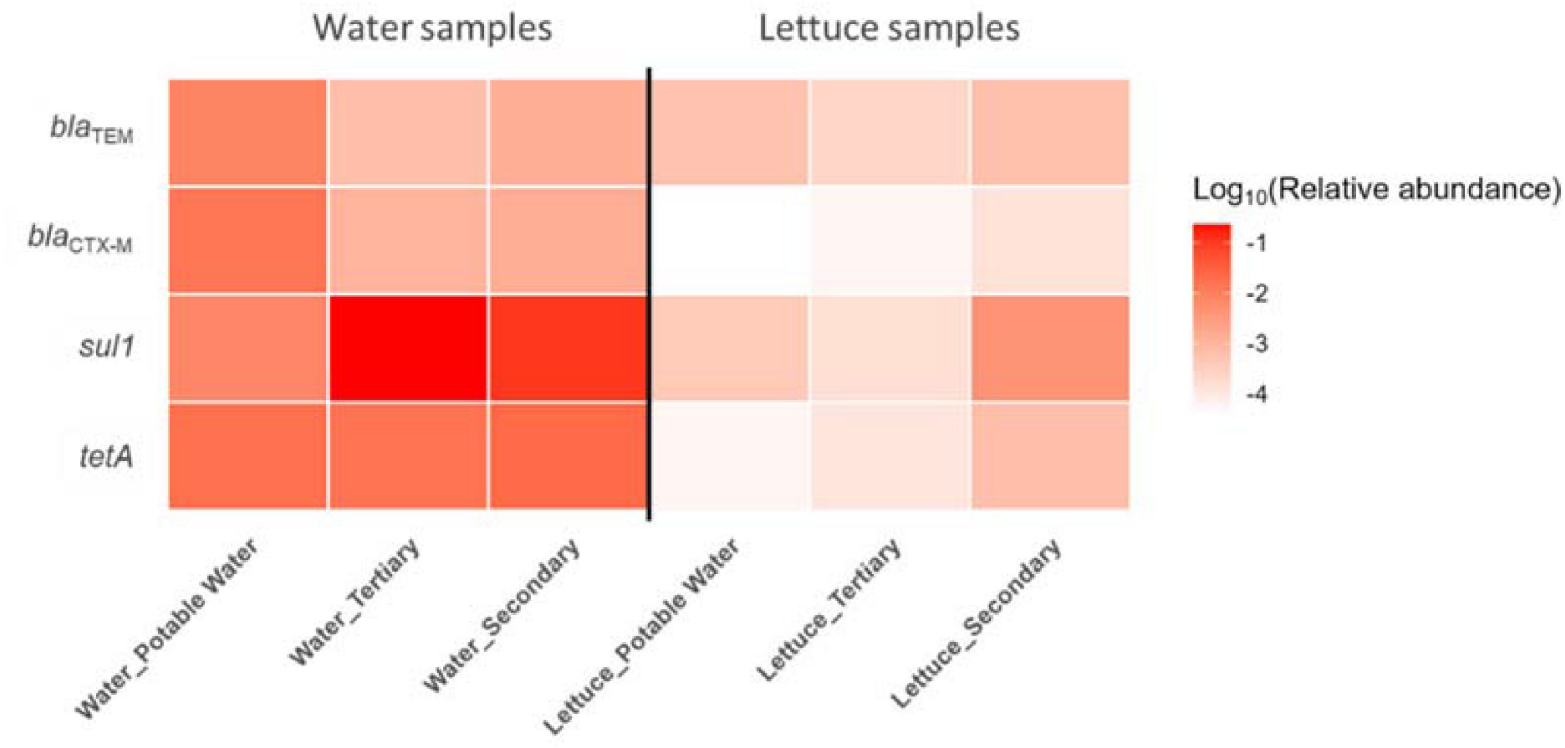
Heatmap showing the mean relative abundances of four antibiotic resistance genes (ARGs) detected in irrigation water and lettuce. Column labels indicate water quality (potable, secondary-treated, tertiary-treated). Color intensity represents log-transformed relative gene abundance (ARG copies per 16S rRNA gene copies).

The relative abundance of each target ARG was calculated as log_10_ (ARG/16S rRNA), and the results for the four genes are shown in Figure 6. Secondary-treated water showed the highest relative abundances of *sul1* and *tetA* genes, followed by tertiary-treated water, while potable water consistently displayed the lowest values. In lettuce, the same pattern was observed, although values were lower than in the corresponding irrigation water. Across both matrices, s*ul1* and *tetA* showed highest relative abundances, whereas *bla*_TEM_ and *bla*_CTX–M–1_ showed the lowest. Overall, secondary- and tertiary-treated water showed higher relative abundances of ARGs than potable water. In lettuce, the same genes were detected, but at lower relative abundances than in the corresponding irrigation water, suggesting that ARGs present in reclaimed water could be transferred to the crop.

## 4 Discussion

In this study, the occurrence of *E. coli* and ESBL-producing *E. coli*, together with the transfer of ARGs, was evaluated throughout the entire growth cycle of baby lettuce, from the seedling stage to commercial maturity. This approach enabled us to compare the potential of different water sources to introduce fecal indicator bacteria and antimicrobial resistance determinants into the crop.

According to our results, previous studies have also shown the efficacy of tertiary treatments to reduce fecal indicators and ARB to undetectable levels (Zurita and White, 2014; Cadena-Aponte et al., 2025; Oliveira et al., 2023; Truchado et al., 2025). In contrast, the persistence of ESBL-producing *E. coli* in secondary treated water confirms that biological treatment alone is insufficient to fully remove bacteria, suggesting secondary effluents as a potential reservoir of ARB (Vital et al., 2018; Yin et al., 2019). Although the concentrations detected were lower than those typically reported in untreated wastewater, secondary-treated water still represents a relevant reservoir of AMR. From a food-safety perspective, these findings underscore the need for tertiary or advanced treatments when irrigating produce intended for raw consumption, as secondary-treated water, despite partial treatment, remains a possible route for introducing both fecal and resistant bacteria into crops (Heyde et al., 2025; Truchado et al., 2025).

The results obtained from the lettuce plants confirm the impact of water treatment efficiency on downstream contamination, as insufficiently treated effluents can transfer residual fecal bacteria to crops (Nüesch-Inderbinen et al., 2015; Summerlin et al., 2021; Niquice-Janeiro et al., 2024).

The observed 16S rRNA gene abundances indicate that the bacterial load in irrigation water strongly depends on the water source, although this does not directly translate into increased bacterial colonization on lettuce surfaces. Consistent with previous studies, our results show that treated effluents retain detectable bacterial DNA even after advanced treatment, depending on process stringency (Ghosh et al., 2021; Seyoum et al., 2022). The > 3-log difference between potable and treated wastewater indicates that treatment markedly reduces total bacteria. In contrast, lettuce irrigated with different water sources showed no significant differences in 16S rRNA gene abundance, suggesting that phyllosphere colonization is driven mainly by leaf surface properties, UV exposure, and native microbial competition rather than by the microbiota present in irrigation water (Machado-Moreira et al., 2021).

Although only four ARGs were analyzed, the selected targets (*bla*_TEM_, *bla*_CTX–M–1_, *tetA*, and *sul1*) represent key antibiotic classes and have been identified as core AMR surveillance markers (EFSA Panel on Biological Hazards (BIOHAZ) et al., 2021; Keenum et al., 2022). In the irrigation water samples used in this study, *sul1* and *tetA* were consistently more abundant, whereas *bla*_TEM_ and *bla*_CTX–M–1_ occurred at lower and more variable levels across treatments (Ghosh et al., 2021; Truong et al., 2021). These water profiles were comparable to those previously reported in agricultural contexts (Sanganyado and Gwenzi, 2019; Chen et al., 2020; Seyoum et al., 2022; Truchado et al., 2025). In contrast, β-lactam resistance markers (*bla*_CTX–M–1_*, bla*_TEM_) typically occur at lower abundances (Cacace et al., 2019).

Under controlled growth-chamber conditions, the transfer of ARGs to lettuce tissues appeared limited. Still, lettuce irrigated with secondary-treated wastewater showed a trend toward higher prevalence and concentration of these genes compared with plants irrigated with potable or reclaimed water. The basal detection of *sul1* and *tetA* in seedlings (starting material) suggested a pre-existing reservoir in the plant or substrate, while the subsequent detection of *bla*_CTX–M–1_ and *bla*_TEM_ in lettuce plants irrigated with secondary-treated water or tertiary-treated water, but not with potable water, indicates irrigation water as a plausible source of these genes. ARG normalization to the 16S rRNA gene showed similar patterns across treatments, indicating that differences reflected changes in the proportion of resistant bacteria rather than total bacterial load.

Our observations are partly consistent with previous studies reporting no substantial increase in ARG loads in plant tissues irrigated with treated wastewater. In spinach and lettuce cultivated under both greenhouse and open-field conditions, Ofori et al. (2025) found no differences in leaf ARG levels between plants irrigated with drinking water and those receiving treated wastewater. Likewise, studies on lettuce (Marano et al., 2019; Shamsizadeh et al., 2021) and tomato (Seyoum et al., 2022) reported no increases in ARGs when treated effluents were used. On a broader scale, long-term field studies such as that of Liu et al. (2022) have shown that environmental and management factors, particularly soil characteristics and contaminants such as heavy metals, can exert stronger influences than water type on resistome dynamics.

Conversely, several studies have reported clear evidence of ARG transmission associated with irrigation. Soler et al. (2025) reported the dissemination of ARGs from treated wastewater effluents to soils and leafy vegetables under field conditions, particularly when microbial loads in the water were high. Araújo et al. (2017) detected ARB and ARGs in edible tissues of leafy greens irrigated with untreated or secondary-treated wastewater, but not with well water, demonstrating direct resistance transfer. In controlled greenhouse experiments, Shen et al. (2019) showed that poor water quality and sprinkler irrigation increase ARG transfer, while Gekenidis et al. (2021) found similar transmission from reservoir water to leafy crops in the field. Overall, meaningful ARG transfer occurs mainly when water quality is low, microbial loads are high, or irrigation brings water into direct contact with leaves.

A key factor explaining differences across studies is the degree of wastewater treatment. In our experiment, although the irrigation waters differed in their ARG content, no differences were observed in lettuce irrigated with potable or tertiary-treated water. These findings are consistent with Ho et al. (2025), who compared potable water with reclaimed water treated through filtration, UV disinfection, and more advanced multi-stage processes (ceramic ultrafiltration, ozonation, and biologically activated carbon) and found that plant ARG uptake declined substantially as treatment complexity increased. In contrast, lettuce irrigated with secondary-treated water in our study showed a trend toward higher concentrations of several resistance genes, underscoring the need for additional treatment steps to ensure the microbiological safety of such water and support its safe reuse in agriculture.

A limitation of this study is that all experiments were conducted under controlled growth-chamber conditions, which, while minimizing environmental variability and enabling a clearer evaluation of irrigation water quality effects, may not fully capture the complexity of field conditions. Factors such as rainfall events, soil–plant–microbe interactions, and potential contamination from surrounding environments could influence ARG dynamics in open-field cultivation. Furthermore, the study focused on short-term accumulation during a single growing cycle and on a limited set of resistance genes; the long-term fate of ARGs in soil and their potential integration into plant-associated microbial communities remain poorly understood. Future research should address these aspects through multi-season field trials, incorporating different crop types and irrigation regimes, and using metagenomic approaches to better understand the diversity, persistence, and potential mobility of ARGs introduced through reclaimed water irrigation.

## 5 Conclusion

In this study, the detection of *E. coli* and ESBL-producing *E. coli* was restricted to plants and secondary treated water, whereas neither potable water nor tertiary water yielded positive results. This microbial pattern was consistent with the genetic data: although secondary treated wastewater contained higher concentrations of ARGs, transfer into lettuce was generally low, and only *tetA* showed statistically significant differences among treatments. The absence of differences between potable water and tertiary treated water, both in bacterial detection and in ARG accumulation, suggests that these sources can be considered safe concerning resistance transfer under controlled conditions.

In contrast, irrigation with secondary treated water was associated with the introduction of cultivable ARB and with a trend toward higher ARG abundances, indicating that this type of water would require additional treatment to meet safety standards for agricultural reuse, particularly when irrigating fresh produce that is consumed raw. Taken together, these findings indicate that although the introduction of resistance from irrigation water into crops is possible, the risk is substantially reduced when higher-quality water sources, such as tertiary water, are used. Nonetheless, the basal detection of certain genes in initial seedlings, and the limited magnitude of treatment-related differences highlight that additional factors, including gene-specific persistence, substrate properties, and plant–microbiota interactions, that also influence resistance dynamics in lettuce.

## Data Availability

The data generated and analyzed in this study are included in the article and its supplementary material. Additional data are available from the corresponding author upon reasonable request.
